# Describing compassion fatigue from the perspective of oncology nurses in Durban, South Africa

**DOI:** 10.4102/hsag.v24i0.1279

**Published:** 2019-10-15

**Authors:** Dorien Wentzel, Anthony Collins, Petra Brysiewicz

**Affiliations:** 1 School of Nursing and Public Health, Nursing University of KwaZulu-Natal, Durban, South Africa; 2 School of Fine Art, La Trobe University, Melbourne, Australia

**Keywords:** compassion fatigue, intervention, oncology nurses, self-care, support

## Abstract

**Background:**

Caring for cancer patients can take a toll on the emotional health of oncology nurses, which may lead to compassion fatigue, resulting in decreased quality of nursing care, absenteeism and decreased retention of staff.

**Aim:**

The aim of this study was to describe compassion fatigue from the perspective of oncology nurses. This study is part of a larger mixed-methods action research study to develop an in-facility intervention to manage compassion fatigue in oncology nurses.

**Setting:**

This study was conducted at Durban, KwaZulu-Natal, South Africa.

**Methods:**

The research setting comprised one state hospital (with oncology clinics and wards), a private hospital (with oncology wards) and a hospice in Durban, KwaZulu-Natal, South Africa. Semi-structured individual interviews (guided by Figley’s Compassion Fatigue Process, 2005) were conducted with eight participants. Data were analysed using manifest content analysis.

**Results:**

Five categories emerged from the data, namely, emotional connection, emotional fatigue, emotional loss, blurring boundaries and acceptance.

**Conclusion:**

The findings revealed that oncology nurses are affected emotionally in caring for their patients, thus making them prone to compassion fatigue. Oncology nurses need to acknowledge compassion fatigue and be able to self-reflect on how they are managing (both positively and negatively) with the stressors encountered in the oncology wards or units.

## Introduction and background

Because of prolonged and continual contact with recurrent deaths, grief and hopelessness experienced by patients and their families, the oncology nurse is at a high risk of developing compassion fatigue (Coetzee & Klopper 2010; Gillespie 2013; Potter et al. 2013). Although nursing cancer patients can provide personal and intellectual fulfilment, this can take a toll on the oncology nurses’ physical and emotional health (Gillespie 2013). The physical and emotional demands of the patient–nurse relationship, the oncology unit, together with daily challenges of nursing patients diagnosed with cancer, can expose oncology nurses to severe stress (Gillespie 2013; Potter et al. 2013). Nurses commonly empathise with patient deaths and may experience a personal sense of futility or failure in their nursing care (Potter et al. 2013; Slocum-Gori et al. 2011). The enduring and repeated patient losses and caring for bereaved families, together with caring for patients with life-threatening illnesses, experienced by oncology nurses predispose them to psychological stress (Gillespie 2013; Potter et al. 2013).

Compassion fatigue is explained as a situation of emotional fatigue stemming from encounters with compassion stress and can rise abruptly without any warning, leaving the nurse feeling confused, secluded and helpless (Figley 2005). Figley (2005) further elaborated that compassion fatigue involved obsession with patients’ collective emotional and physical sufferings, which resulted in emotional stress. Figley (1995) softened the term secondary traumatic stress disorder (STSD) to the user-friendly expression compassion fatigue, and associated it with compassion fatigue among health care practitioners in clinical practice who are first to witness pain, suffering and distress. Compassion fatigue has frequently been referred to as the ‘cost of caring’ (Figley 2005). Nurses who are suffering from compassion fatigue can still care for their patients; however, their care may be less compassionate (Slocum-Gori et al. 2011). Symptoms of compassion fatigue include physical, mental and emotional exhaustion, disconnection and depersonalisation, and isolation from peers, together with a lessened sense of personal achievement (Figley 2005). Adverse effects of compassion fatigue are an increase in staff turnover and absenteeism, a decrease in the quality of patient care, decreased patient satisfaction and patient safety, and it can have a significant effect on the healthcare professionals’ personal life (Boyle 2011).

Management and health care practitioners are aware of the occupational stress experienced by health care practitioners; however, there is confusion concerning the formal definitions of burn out, secondary stress syndrome (secondary stress in traumatology), secondary victimisation, secondary traumatic stress, secondary survivor, compassion fatigue and vicarious traumatisation (Boyle 2011; Coetzee & Klopper 2010). Compassion fatigue has been proposed as a replacement for STSD; however, Coetzee and Klopper (2010) disagree, as they believe it does not encompass the fundamental meaning of compassion fatigue.

In South Africa, there is currently an increase in cancer patients and survivors and/or remission, together with an increase in HIV-related cancers, which further burdens overwhelmed oncology nurses. Because of the increase in cancer patients, there is an increased demand for health care resources (Moten, Schafer & Ferrari 2014), including drugs, radiotherapy, hospitals, hospices, oncologists and oncology nurses. As advances in cancer treatment have increased dramatically, because each type of cancer is diverse from the other and treatment modalities are different for each type, and more patients are surviving cancer, there is a need for competent, empathetic and up-to-date nursing care (Moten et al. 2014).

The aforementioned factors exert additional pressure on oncology nurses to be able to care for their patients with compassion, which could predispose them to developing compassion fatigue (Potter et al. 2013).

## Statement of research problem

Nurses in South Africa are confronted with ‘unbearable workloads, poor working conditions, and lack of resources’, which negatively impact their physical and psychosocial well-being (Knobloch 2007:7). Oncology nursing is a worthwhile and gratifying profession; however, exposure to highly stressful incidents can affect psychological well-being (Zander, Hutton & King 2010). Oncology nurses are prime candidates for compassion fatigue because of their extended compassionate assistance to patients and their families, repeated exposure to patients experiencing trauma from the aggressive side-effects of cancer treatments and the severe symptoms experienced in the (Potter et al. 2013). This study therefore aimed at describing compassion fatigue from an oncology nurses’ perspective.

## Aim of the study

The aim of this study was to describe compassion fatigue from the perspective of oncology nurses practising in oncology departments in Durban, KwaZulu-Natal, South Africa. This study is part of a larger mixed-methods action research study to develop an in-facility intervention to manage compassion fatigue in oncology nurses in Durban, KwaZulu-Natal, South Africa.

## Definitions of key concepts

Compassion fatigue: It is defined as ‘a state of exhaustion resulting from exposure to those suffering from the consequences of traumatic events’ (Figley 1995:17). It is a natural result of working with patients who are experiencing stressful events, and occurs because of the excessive energy and compassion given over a protracted time to patients who are suffering. The helpers, in offering empathy and compassion, become immersed in their patients’ pain and trauma, and, in turn, become traumatised (Figley 2005).

Nurses working in oncology wards: Registered professional nurses and nurses enrolled with the South African Nursing Council, with or without further oncology qualifications, who are currently employed in oncology units and wards/hospices and have a minimum of 6 months experience in caring for oncology patients in Durban, KwaZulu-Natal.

## Research methodology

This study is part of a major study that used action research with a mixed-methods sequential explanatory design. An exploratory qualitative approach, using in-depth interviews, and manifest content analysis assisted the researchers in their exploration into compassion fatigue from the perspective of nurses working in oncology wards (Erlingsson & Brysiewicz 2017; Graneheim & Lundman 2003).

### Research setting and participants

This study was conducted in three settings in the Durban metropolitan area of South Africa. The settings included a hospice (non-government organisation [NGO]), oncology clinics (within a state hospital) and oncology wards (within state and private hospitals). The different settings were chosen because they reflect the medical continuum of cancer from diagnosis to death, and they represent the majority of cancer nursing care in Durban from the public and private sectors and an NGO:

- Site 1 is a hospice (NGO) in Durban where patients receive palliative care from nurses, either in the patients’ homes or in the in-patient unit.
- Site 2 is a public–private partnership institution in Durban, which comprises oncology clinics and five in-patient wards for oncology patients. In the clinics, patients are seen on an outpatient basis and receive chemotherapy and/or radiation therapy. In the in-patient wards, patients are admitted overnight, or for longer, and receive chemotherapy, radiation and palliative care, as well as undergoing various medical and surgical procedures related to the cancer diagnosis.
- Site 3 is a private institution for patients with private health insurance. This site has two in-patient oncology units consisting of an acute unit and a step down/palliative unit. Both oncology units admit patients for chemotherapy, radiation and palliative care.

To identify all nurses (including management) working in the oncology wards of the three settings, purposive sampling was utilised. The inclusion criteria included professional nurses (registered with 4 years of education and training) and enrolled nurses (with 2 years of general nursing education and training) with or without additional oncology/palliative care education and training; participants were selected based on results from the quantitative portion of the larger study (Wentzel & Brysiewicz 2018). The participants needed to have worked in one of the three oncology settings for a minimum of 6 months.

### Data collection process

Data were collected over 5 months, from February to June 2017. Once permission was obtained from the individual institutions, communication with unit managers of the three institutions was established to discuss the purpose and objectives of the proposed study, and to request permission to access the oncology nurses. Dates and times were arranged with participants for interviews. On the request of the nurses, and with permission of the management, interviews were conducted in a private room during protected time, which was usually kept for in-service training, to ensure that patients’ care was not affected. D.W. found that the interviews revealed a great deal about how nurses felt and their experiences of physical and emotional fatigue in caring for cancer patients, and was able to probe for emotional and psychological stressors, protective mechanisms and resources used to manage compassion fatigue. Because of the prolonged interaction with participants, the researcher did not experience any concerns regarding the clarity of the questions posed to the participants.

Before commencement of the interviews, the researcher obtained written informed consent. Each interview, which was conducted in English, lasted approximately 45–60 min. The interview questions were guided by the Compassion Fatigue Process (Figley 2005) and included ‘What do you understand by compassion fatigue?’ ‘What emotional and psychological stresses contribute to compassion fatigue?’ After interviewing eight participants and analysing the data, all three researchers concluded that redundancy and saturation of data had been reached.

### Data analysis

Analysis was conducted using manifest content analysis (descriptive analysis), which includes questions about who, what, when or where? (Erlingsson & Brysiewicz 2017; Graneheim & Lundman 2003). All interviews were recorded and transcribed verbatim, and a database of participants’ transcripts was compiled and saved on a computer protected by a password known only to the researchers. The data were read and re-read, then manually analysed to uncover meaning units, which were then condensed, coded and grouped into specific categories (Erlingsson & Brysiewicz 2017) (see Table 1).

**TABLE 1:** Example of content analysis coding and categorisation.

Meaning unit	Condensation	Code	Category
They creep into your heart	Creep into your heart	Affects emotionally	Emotional connection
Fatigue you get from, for caring patients, especially those who are dying	Fatigue from caring	Fatigue	Emotional fatigue
If they have to pass … it takes a bit of you … it takes a small piece of you	Takes a bit of you	Takes pieces	Emotional loss
You take it home with you, it’s kept in your mind	Kept in your mind	No off switch	Blurring boundaries
I’m at that stage of acceptance, that it was inevitable … kind of pacified myself in saying that this patient is no longer suffering	Stage of acceptance	Accepting	Acceptance

### Rigour

The researcher was guided by the four criteria of trustworthiness as proposed by Guba and Lincoln (1994). By spending prolonged time with nurses working in the oncology wards for approximately 2 years, a good rapport was created with them. The participants were asked to be ‘open and free’ during the interviews to collect useful, rich and thick descriptions, thus achieving credibility. All three researchers were involved in the data analysis process, with two of the researchers (research supervisors) having expertise in qualitative research and one in psychology (A.C.). Two researchers (D.W. and P.B.) independently reviewed the data, which were then discussed and verified with the codes and categories by all three researchers, thereby demonstrating confirmability. Participants also reviewed the categories and agreed that they were a true representation of their descriptions or perceptions. Participants did not suggest any changes, thereby confirming the findings. The researcher provided a detailed methodological description, and an audit trail was used to establish dependability (Guba & Lincoln 1994). To address transferability, the researcher strove to provide thick descriptions to allow the reader to decide if these study findings could be transferred to their own setting (Shenton 2004).

### Ethical considerations

The research commenced after receiving ethical clearance from the University Ethics Committee (BF140/14), from the three institutions (state and private), from the KwaZulu-Natal Department of Health Research Unit and from Hospice Palliative Care Association of South Africa. The research could possibly evoke recalling of emotionally distressing incidents, so the researcher (D.W.) negotiated with the employee assistance programmes at the three settings regarding support available for participants who may need referral; three participants were referred. Prior to each interview, the aim of this study was explained, and using a participant information sheet written informed consent was obtained. Permission to audio-record the interview was obtained from the participants. Participants were assured that participation was voluntary and that they could withdraw from the study with no repercussions. In order to ensure that the data obtained cannot be traced back to the participants, pseudonyms were used to ensure confidentiality.

## Findings

### Participant demographics

All the participants were females, aged between 21 and 57 years (average age of 37 years), with between 2 and 27 years of work experience in oncology wards. Participants’ qualifications included two enrolled nurses on day duty, one professional nurse on night duty and two professional nurses on day duty, and three unit managers of oncology units. Two participants had an added oncology qualification and one participant had a palliative care qualification.

Five categories emerged from the data, namely, emotional connection, emotional fatigue, emotional loss, blurring boundaries and acceptance.

### Emotional connection

Because of the long and extensive nature of the management of cancer, patients spend a lot of time in the health care facilities receiving chemotherapy, radiation, surgery and care for complications. These prolonged and recurrent visits to health care facilities foster good and reciprocal relationships with oncology nurses. Excerpts from oncology nurses reinforced the close emotional relationship formed between nurses and their patients:

‘We have patients who have been with us for so long, they become part of your family.’ (Reshma, female, 36 years old)

‘You build a relationship with them, you know them and they know about your family.’ (Jane, female, 23 years old)

‘We have relationships with the family … we are part of the family.’ (Sweetlips, female, 35 years old)

‘You get to know your patients; we get so close to patients.’ (Rajayshri, female, 46 years old)

The participants went on further to describe:

‘… [*T*]hey creep into your heart.’ (Veronica, female, 57 years old)

‘Children have a way of creeping into your heart.’ (Candidate X, female, 45 years old)

### Emotional fatigue

All the participants described feeling extremely tired. However, the tiredness was not only physical tiredness but also emotional tiredness.

One participant illustrated this:

‘It’s tiredness … but not physical as such … but it’s emotional and mental.’ (Sweetlips, female, 35 years old)

Sumeshni (female, 33years old) explained further:

‘Fatigue from within … from caring.’

Thoba (female, 33 years old) reinforced:

‘So that’s the fatigue we feel fatigue from within … from caring.’

Lauren (female, 29 years old) echoed this by explaining how she became physically and emotionally involved in caring for her patients:

‘Giving my all for the patients … encountering feeling for them.’

### Emotional loss

Participants described the deep personal loss they felt after the death of their cancer patients. They elaborated how they experienced this emotional loss.

Two participants stated:

‘If they [*the patient*] have to pass on … it takes a bit of you … it takes a small piece of you.’ (Charlene, female, 51 years old)

‘When we lose a patient … especially one that has been with us a long time … we do get affected … it’s kept in your mind.’ (Sumeshni, female, 33 years old)

Sasha (female, 21 years old) explained what this did to her:

‘It takes too much out of you.’

### Blurring boundaries

Participants agreed it was hard not to take ‘their patients and their work’ home with them, and they had difficulty in separating their professional and personal lives as it was ‘not easy to switch off’.

Devi (female, 41 years old) said:

‘It takes toll on your personal life.’

Another elaborated:

‘When you go home, you go home with the stuff that is happening at the hospital, you worry about the patients … not easy to switch off.’ (Sweetlips, female, 35 years old)

Another participant depicted her difficulty in separating her professional and personal life:

‘I try not to take too much with me … I also try to separate the two … but you can’t cut completely.’ (Thoba, female, 33 years old)

Lauren (female, 29 years old) explained how this affected her and resulted in a lack of empathy at home:

‘I’m not as empathetic in my home life as I could be … because my mind is ongoing with the problems that I have had at work.’

### Acceptance

Despite the inevitably of the patients’ outcomes, participants placated themselves that they had to accept that ‘life must go on’ and they should ‘move on’:

‘We are so used to everything that happens … at the back of my head, I knew that this would happen.’ (Thoba, female, 33 years old)

‘I’m at that stage of acceptance, that it was inevitable … kind of pacified myself in saying that this patient is no longer suffering.’ (Sasha, female, 21 years old)

‘So what must be, must be … this is the nature of our job.’ (Lauren, female, 29 years old)

Participants admitted that over time, the deaths of their patents forced them to become stronger and to come to terms with the reality, which prepared them for the next death:

‘In time, it makes you grow, you learn from previous [*deaths*] and you grow. You learn from it … and you talk about it … and you HAVE to move on …It makes you learn and grow and helps you for the next one … that’s how we cope.’ (Devi, female, 41 years old)

‘It [*deaths*] makes you stronger.’ (Charlene, female, 51 years old)

## Discussion

This study described compassion fatigue from oncology nurses’ perspectives. Categories that emerged from the data included emotional connection, emotional fatigue, emotional loss, blurring boundaries and acceptance.

Because of the numerous hospitalisations, oncology nurses have increased opportunities to develop empathetic connections and trust with their patients (Rohani, Kesbakhi & Mohtashami 2018). Listening empathetically to their patients promotes patients’ self-disclosure, which improves emotional connection and ultimately patient outcomes (Rohani et al. 2018). Participants spoke positively about forming emotional connections with their patients. Wittenberg-Lyles, Goldsmith and Reno (2014) and Boyle (2011) noted that emotional connections can develop when staff experience close emotional attachments to patients, and this can lead to over-identification with the patient without foreseeing the emotional consequences.

Maja (2016) concurs with this study’s findings that nurses, reportedly overwhelmed by emotional burdens and the deaths of patients, described themselves as feeling physically and emotionally exhausted, expressing it as ‘fatigue from within’. Researchers suggest that this emotional exhaustion experienced by oncology nurses can progress to emotional fatigue and distress (Barbour 2016; Leung et al. 2012). One protective mechanism oncology nurses could develop to overcome feelings of helplessness and the inability to assist patients during their traumatic incidents is self-compassion and self-kindness. Self-compassion is associated with positive psychological traits (namely, well-being and emotional intelligence) and could promote resilience in oncology nurses and have an influence on patient satisfaction (Duarte & Pinot-Gouveia 2017). Wolf et al. (2015) suggest that this deep emotional fatigue is a form of unacknowledged moral distress.

Participants described the feelings of deep personal loss following the death of their oncology patients, expressing it as ‘it takes a bit of you’. Slocum-Gori et al. (2011) concur that nurses who are unable to assist their dying patients frequently experience loss of self. This emotional loss can result in nurses harbouring a sense of distress and anxiety, with the consequent emotional turmoil that can lead to loss of self-efficacy, loss of self professionally and loss of self personally (Duarte & Gouveia 2017). Because of the traumatic nature of caring for cancer patients, oncology nurses frequently have negative and intrusive thoughts, and in an attempt to cope with these thoughts, they resort to detachment and avoidance strategies to reduce the effect of difficult internal experiences – a response pattern referred to as psychological inflexibility (Duarte & Pinto-Gouveia 2017). Although providing short-term relief, psychological inflexibility could become psychologically maladaptive (Duarte & Pinto-Gouveia 2017). It is important to note that distancing and de-personalisation from the emotional requirements of patients lead to a lack of empathy in caring, which eventually increases the risk of developing compassion fatigue (Maja 2016). Boyle (2011) describes this emotional disconnection as a response nurses employ to cope with emotional attachments to patients. Contrary to the findings in this current study, it is noted that repeated and constant exposure to misfortune renders some nurses unable to detach from their patients. Detachment and over-identification represent two extremes in coping responses that oncology nurses may employ; however, this also highlights the tension the oncology nurse must deal with, namely, is there a risk of over-identifying with the patient or does one prevent oneself from over-identifying? Oncology nurses frequently feel helpless and vulnerable, and resort to self-blame when they witness patients in intractable pain, or when patients die (Maja 2016). Nolte et al. (2017) explain the notion of loss of self as encompassing feelings of emotional disablement and isolation, coupled with a sense of professional and personal failure. A suggestion for coping with loss of self is to promote psychological flexibility – the ability to encompass the negative experiences (Duarte & Pinto-Gouveia 2017). Oncology nurses who display psychological flexibility are concerned about their patients’ suffering and are able to encompass negative experiences associated with seeing their patients’ distress (Duarte & Pinto-Gouveia 2017).

Wittenberg-Lyles et al. (2014) support this study’s findings that emotional stressors encountered at work are often relived at home, influencing personal relationships and resulting in sleeplessness and fatigue. Maja (2016) suggests that over-identifying with a patient reduces nurses’ ability to balance their professional and personal life. Kushnir Talma, Stanley and Azulai (1997) state that long hours at work, coupled with the inability to set professional and personal boundaries, cause emotional exhaustion in oncology nurses. Leung et al. (2012) reiterate the importance of being able to delineate one’s professional and personal boundaries to cope with existential concerns encountered in the care of cancer patients, and maintaining a professional boundary may help oncology nurses to develop empathy with their patients’ distress.

Participants described the inevitable outcome for their patients by voicing ‘life must go on’. Flarity et al. (2013) stated that nurses are motivated to be emotionally strong to project a professional image of being able to cope with whatever situations they may come across. This imposed motivation to adhere to hospital culture and display stoicism could exacerbate the development of compassion fatigue (Brint 2017; Flarity et al. 2013). Participants acknowledged that the deaths of their patients compelled them to come to terms with it and move on. Contrary to the study’s findings, Kubler-Ross (1969) proposed that part of the acceptance process is to remember, internalise and come to terms with patients’ diagnoses and eventual death.

## Limitations

The limitations of this study were that all the participants were female, it was conducted in only three settings in Durban and the researcher was known to some of the participants, which may have influenced them to provide socially acceptable answers.

## Conclusion

Because of the numerous physical and emotional demands of nursing cancer patients, there is currently concern regarding oncology nurses potentially succumbing to psychological stress, thereby putting themselves at risk of developing compassion fatigue. Nurses working in oncology wards described compassion fatigue, the stresses encountered in these settings, emotional connections, emotional fatigue, emotional loss, blurring of boundaries and acceptance while nursing oncology patients. The findings from this study support the fact that oncology nurses are affected in many ways when caring for their patients, making them prone to compassion fatigue.

## Recommendations

This study’s findings demonstrated the need to equip nurses working in oncology wards with the necessary knowledge and skills to recognise and manage compassion fatigue and burn out. Institutions should provide regular professional educational programmes to assist nurses to cope with the emotional demands of nursing. Institutional management should be aware of the possibility of their oncology nurses developing compassion fatigue, and policies, guidelines and strategies should be in place to offer psychological support to oncology nurses.
